# Transcriptome-Wide m6A Methylation Landscape of Longissimus Dorsi Muscle in Indigenous Guizhou Cattle

**DOI:** 10.3390/life16081252

**Published:** 2026-07-29

**Authors:** Junda Wu, Qingyun Huang, Xin Wang, Xiaoping Wei, Bo Yu, Rong Yang, Longxin Xu, Yongcai Zhu

**Affiliations:** 1 Institute of Animal Husbandry and Veterinary Science, Guizhou Academy of Agricultural Sciences, Guiyang 550005, China; 13621242537@163.com (J.W.);; 2 Guizhou Provincial Key Laboratory of Livestock and Poultry Genetic Resources Innovation and Utilization, Guiyang 550005, China; 3 Guizhou Provincial Breeding Livestock and Poultry Germplasm Determination Center, Guiyang 550018, China

**Keywords:** guizhou cattle, longissimus dorsi muscle, N6-methyladenosine, m6A modification, MeRIP-seq, skeletal muscle, meat quality

## Abstract

Guizhou cattle are important indigenous bovine genetic resources for regional beef production and germplasm conservation. This study characterized the transcriptome-wide N6-methyladenosine (m6A) enrichment landscape in longissimus dorsi muscle from three representative indigenous Guizhou cattle breeds—*Guanling (GL)*, *Wuchuan (WC)*, and *Weining (WN)*—and compared these profiles with those of *Simmental (XM)* cattle as a commercial reference. MeRIP-seq was used to detect group-level m6A-enriched regions and regions with differential m6A enrichment, followed by Gene Ontology, Kyoto Encyclopedia of Genes and Genomes, and database-inferred protein–protein interaction analyses. qRT-PCR was used to examine transcript abundance of selected network-prioritized candidate genes, rather than to validate m6A enrichment. MeRIP-seq detected 17,659, 19,530, 17,501, and 16,271 m6A-enriched regions in GL, WC, WN, and XM cattle, respectively. Compared with XM cattle, 2822, 5914, and 3655 regions with differential m6A enrichment were detected in GL, WC, and WN cattle, respectively. *ACTB, CTNNB1*, and *AKT2* were prioritized for follow-up, and qRT-PCR showed breed-associated differences in their transcript abundance. These exploratory findings provide an epitranscriptomic resource and candidate targets for future MeRIP-qPCR, phenotypic, and functional studies.

## 1. Introduction

Guizhou cattle are important indigenous bovine genetic resources in southwestern China. *Guanling, Wuchuan, Weining, Sinan*, and *Liping* cattle are the five major native breeds of Guizhou Province and have undergone long-term natural and artificial selection in mountainous environments [1]. In this study, *Guanling, Wuchuan*, and *Weining* cattle were selected as representative indigenous breeds from distinct ecological regions of Guizhou Province. Their distinct population and breeding backgrounds provide a basis for investigating breed-associated molecular variation in skeletal muscle and for comparison with Simmental cattle as a commercial reference.

Skeletal muscle development is a major biological determinant of beef production performance and meat quality. The longissimus dorsi muscle is closely associated with economically important traits, including muscle yield, tenderness, intramuscular fat deposition, and palatability [2]. Meat quality formation is a complex biological process involving the coordinated regulation of muscle fiber development, energy metabolism, adipogenesis, and postmortem muscle transformation [3]. Previous cattle studies have mainly focused on genomic variation, transcriptomic regulation, and protein expression related to muscle development and meat quality traits. However, the epigenetic and epitranscriptomic mechanisms underlying phenotypic differences among cattle breeds remain poorly understood.

Among epitranscriptomic modifications, N6-methyladenosine (m6A) is a prevalent internal modification in eukaryotic mRNA and participates in post-transcriptional regulation. Its abundance and distribution can vary among RNA classes, tissues, and biological contexts. m6A can influence RNA splicing, stability, transport, degradation, and translation through methyltransferases (\“writers\”), demethylases (\“erasers\”), and binding proteins (\“readers\”) [4]. Evidence for m6A-associated regulation of skeletal muscle development has been obtained from several experimental systems. In cattle, analyses of longissimus dorsi muscle and cultured bovine skeletal myoblasts implicated METTL3, METTL14, FTO, and ALKBH5 in myoblast proliferation and differentiation [5]. A subsequent study combined bovine myoblasts, mouse C2C12 cells, and a mouse muscle-regeneration model to investigate METTL3–YTHDF2-dependent regulation of TM4SF1 [6]. In pigs, developmental m6A profiles were examined in prenatal skeletal muscle, whereas functional analyses of METTL14 and IGF2BP1 were mainly performed in C2C12 cells [7]. MYOD1-related regulation has also been examined in Guanling cattle muscle tissues and a bovine kidney-derived cell line, although that study did not directly investigate m6A [8]. Together, these studies support the relevance of m6A-associated processes to skeletal muscle biology, but direct in vivo evidence in cattle and links to meat-quality phenotypes remain limited.

Simmental cattle are widely used commercial beef cattle worldwide because of their superior growth performance, high feed conversion efficiency, and desirable carcass traits. In China, Simmental cattle and Simmental-derived crossbreeding systems are important genetic resources for commercial beef production because of their rapid muscle growth and stable production performance under standardized management conditions. In contrast, indigenous Guizhou cattle populations have been maintained under local ecological and production conditions, whereas *XM* cattle represent a widely used commercial breed. Comparing these populations provides an exploratory framework for characterizing group-associated variation in skeletal muscle m6A enrichment across distinct genetic and production backgrounds.

Studies on m6A methylation in bovine skeletal muscle remain limited, particularly in indigenous Chinese cattle breeds. Moreover, the potential contribution of m6A modification to breed-associated differences in muscle development and meat quality remains unclear. In this study, methylated RNA immunoprecipitation sequencing (MeRIP-seq) was used to characterize transcriptome-wide regional m6A enrichment profiles in longissimus dorsi muscle from three representative Guizhou cattle breeds: *Guanling*, *Wuchuan*, and *Weining* cattle. The study aimed to identify candidate regions with differential m6A enrichment and their associated genes, thereby providing an epitranscriptomic resource for future functional and phenotype-integrated studies. The identified regions and genes were treated as candidates for subsequent validation rather than as confirmed functional regulators of beef production traits.

## 2. Materials and Methods

### 2.1. Sample Site and Sample Collection

Three longissimus dorsi muscle samples were obtained from each of *Guanling (GL)*, *Wuchuan (WC)*, *Weining (WN)*, and *Simmental (XM)* cattle (Bijie, Guizhou, China). Three longissimus dorsi muscle samples were collected from each cattle breed. For MeRIP-seq, equal amounts of total RNA from the three animals within each breed were pooled before library construction. All animals were healthy 24-month-old bulls reared at the Guizhou Provincial Breeding Bull Station under a unified management program, including a grazing-plus-housing system and conventional diets. GL, WC, and WN cattle were sampled after slaughter at a slaughterhouse in Guizhou Province, whereas XM cattle were sampled at the breeding bull station. Each sample was obtained from a different animal using the same standardized post-mortem procedure. For qRT-PCR analysis, five independent biological replicates were included per breed. Individual feed intake and body condition scores were not recorded. All animal procedures were approved by the Animal Welfare and Ethics Review Committee of the Guizhou Provincial Institute of Animal Husbandry and Veterinary Medicine (Approval No. AWE-GZSXMSY-2025-29; 15 November 2025).

### 2.2. Library Preparation

For each cattle breed, pooled RNA from three animals was used to construct one m6A immunoprecipitation library and one corresponding input library. Thus, the MeRIP-seq libraries represented pooled breed-level samples rather than independently sequenced biological replicates.m6A-modified RNA was profiled by MeRIP-seq at Novogene (Beijing, China). Total RNA (2 μg) was extracted from each longissimus dorsi muscle sample. RNA integrity and concentration were assessed using an Agilent 2100 Bioanalyzer (Agilent, Santa Clara, CA, USA) and a SimpliNano spectrophotometer (SimpliNano, Salem, UT, USA), respectively. The capillary electrophoresis with a QIAxcel Connect (Qiagen, Venlo, The Netherlands) was used to evaluate RNA integrity number (RIN), and RNA samples with a RIN ≥ 8 were used in RNA library constructions. RNA was fragmented to an average length of approximately 100 nt before immunoprecipitation. Fragmented RNA was incubated with an anti-m6A polyclonal antibody for 2 h at 4 °C. Immunoprecipitated and input RNA were used to construct libraries with the Ovation SoLo RNA-Seq System Kit and sequenced in 2 × 150 bp paired-end mode on an Illumina NovaSeq platform. The approximately 100-nt value refers to the RNA fragment length, whereas 2 × 150 bp refers to the sequencing read configuration; reads from short inserts could therefore overlap.

### 2.3. Quality Control

Raw FASTQ reads were processed using fastp v0.19.11 [9] to trim adapter sequences and remove poly-N, and low-quality reads. The reported IP and input libraries yielded 20.69–25.00 million clean reads, with Q20 values of 97.41–97.90% and Q30 values of 92.86–93.74% (Table 1). GC content was also calculated, and all downstream analyses used the resulting high-quality clean reads.

### 2.4. Read Mapping to the Reference Genome

The *Bos taurus* ARS-UCD1.3 reference genome (GCF_002263795.2) and corresponding NCBI annotation were used. Clean reads were aligned using BWA-MEM v0.7.12 with default parameters, consistent with previously reported MeRIP-seq workflows [10,11]. Only uniquely mapped reads were retained; mapping statistics are provided in Table 2.

### 2.5. Peak Calling

m6A peak calling was performed at the pooled breed level using the exomePeak R package (version 2.16.0). For each breed, the pooled IP library and its corresponding input library were analyzed to identify representative m6A-enriched regions. The resulting peak sets were used to summarize breed-level m6A enrichment patterns, including peak number, transcript-feature distribution, and motif enrichment.

### 2.6. Peak Annotation

Peak annotation was performed using exomePeak v2.16.0 with default parameters based on the NCBI ARS-UCD1.3 gene annotation. Peaks overlapping multiple transcript regions were classified according to the default transcript-region annotation implemented in exomePeak, without customized reassignment. Genes overlapping with or annotated to m6A peaks were defined as m6A peak-associated genes and were used for functional enrichment analyses. In addition, the distribution of m6A peaks across annotated transcript regions, including the TSS, 5′UTR, CDS, stop-codon region, and 3′UTR, was summarized based on the default exomePeak annotation.

### 2.7. Differential m6A Peak Analysis

Differential m6A peak analysis was performed using the exomePeak R package (version 2.16.0). *p*-values generated by exomePeak were corrected for multiple testing using the false discovery rate (FDR) method. Differentially enriched m6A peaks were identified using FDR < 0.05 and FC = 1. The FC cutoff was set to 1 in the analysis pipeline, and the direction of m6A enrichment was used to classify hypermethylated and hypomethylated peaks. Genes associated with differentially enriched m6A peaks were used for subsequent GO, KEGG, and PPI analyses. To further compare m6A methylation profiles among the three indigenous Guizhou cattle breeds, pairwise differential m6A peak analyses were performed among GL, WC, and WN cattle. Normalized m6A enrichment signals of differentially enriched peaks from the WN vs. WC, WC vs. GL, and WN vs. GL comparisons were extracted and visualized as heatmaps. Motif enrichment analysis was performed using HOMER v4.9.1 with default parameters. Motifs were searched on both strands, and no transcript-strand-specific analysis was performed. Enriched motifs were evaluated for compatibility with the canonical RRACH m6A consensus. The top enriched motifs were used to evaluate whether breed-associated differential peak regions contained sequence features compatible with m6A modification.

### 2.8. Database-Inferred PPI Network Construction and Topological Candidate Prioritization

A database-inferred protein–protein interaction (PPI) network was constructed to prioritize genes associated with differentially enriched m6A peaks. All protein-coding peak-associated genes were queried in STRING (Bos taurus, taxid 9913), and interactions with a combined confidence score ≥0.7 were retained. For this revision, selected edges were re-examined in STRING v12.0. STRING scores were interpreted as confidence in functional association rather than interaction strength. Because STRING may integrate direct and orthology-transferred evidence, these associations were not considered cattle-specific experimental validation. The network was visualized in Cytoscape v3.9.1. Degree centrality was used to rank high-connectivity candidate nodes, supported by betweenness, closeness, and eigenvector centrality. These network-prioritized candidates were not considered experimentally validated hubs or evidence of active interactions in the sampled muscle.

### 2.9. qRT-PCR Measurement of Transcript Abundance in Selected Candidate Genes

Longissimus dorsi muscle samples from five independent animals per breed were used for qRT-PCR. Total RNA was extracted according to the manufacturer’s protocol, and concentration, purity, and integrity were assessed before reverse transcription. Complementary DNA was synthesized using a commercial reverse-transcription kit. qRT-PCR quantified steady-state transcript abundance and was not used to measure or validate m6A enrichment. *ACTB*, *CTNNB1*, and *AKT2* were selected for transcript-abundance assessment based on their association with differentially enriched m6A regions, relatively high connectivity in the inferred PPI network, reported relevance to skeletal muscle biology, detectable expression in longissimus dorsi muscle, and primer specificity. These genes represented distinct functional contexts and were not intended to provide an exhaustive assessment of all highly connected network genes.

*ACTB*, *AKT2*, and *CTNNB1* transcript abundance levels were measured using SYBR Green chemistry. *GAPDH*—not *ACTB*—was selected before analysis as the single internal reference gene. Amplification specificity was assessed by melting-curve analysis, and relative abundance was calculated using the 2^−ΔΔCt^ method [12]. Reference-gene stability was not independently evaluated using multiple candidate genes, geNorm, or NormFinder; this limitation is acknowledged. Group differences were assessed using Duncan’s test at *p* < 0.05. Primers are listed in Supplementary Table S1.

## 3. Results

### 3.1. Overall Characteristics of m6A Methylation in the Longissimus Dorsi Muscle

MeRIP-seq generated 20.69–25.00 million clean reads for each reported IP or input library (Table 1). Q20 and Q30 values exceeded 97.4% and 92.8%, respectively. More than 92% of clean reads were mapped to the reference genome, and more than 96% of mapped reads were uniquely aligned (Table 2). These metrics document the sequencing depth and alignment performance used for the group-level analyses.

Annotation of m6A-enriched regions provides essential information for describing the transcriptomic distribution of RNA methylation. RNA fragments enriched by m6A-specific antibodies were subjected to high-throughput sequencing. Peak calling for each group was performed using exomePeak v2.16.0, with FDR < 0.05 and fold enrichment > 1. Based on group-level m6A peak calling, 17,659 m6A peaks associated with 15,269 genes were identified in GL cattle. In WC cattle, 19,530 peaks associated with 16,327 genes were identified. In WN cattle, 17,501 peaks associated with 15,592 genes were identified. In XM cattle, 16,271 peaks associated with 14,675 genes were identified (Table 3).

**Figure 1:**
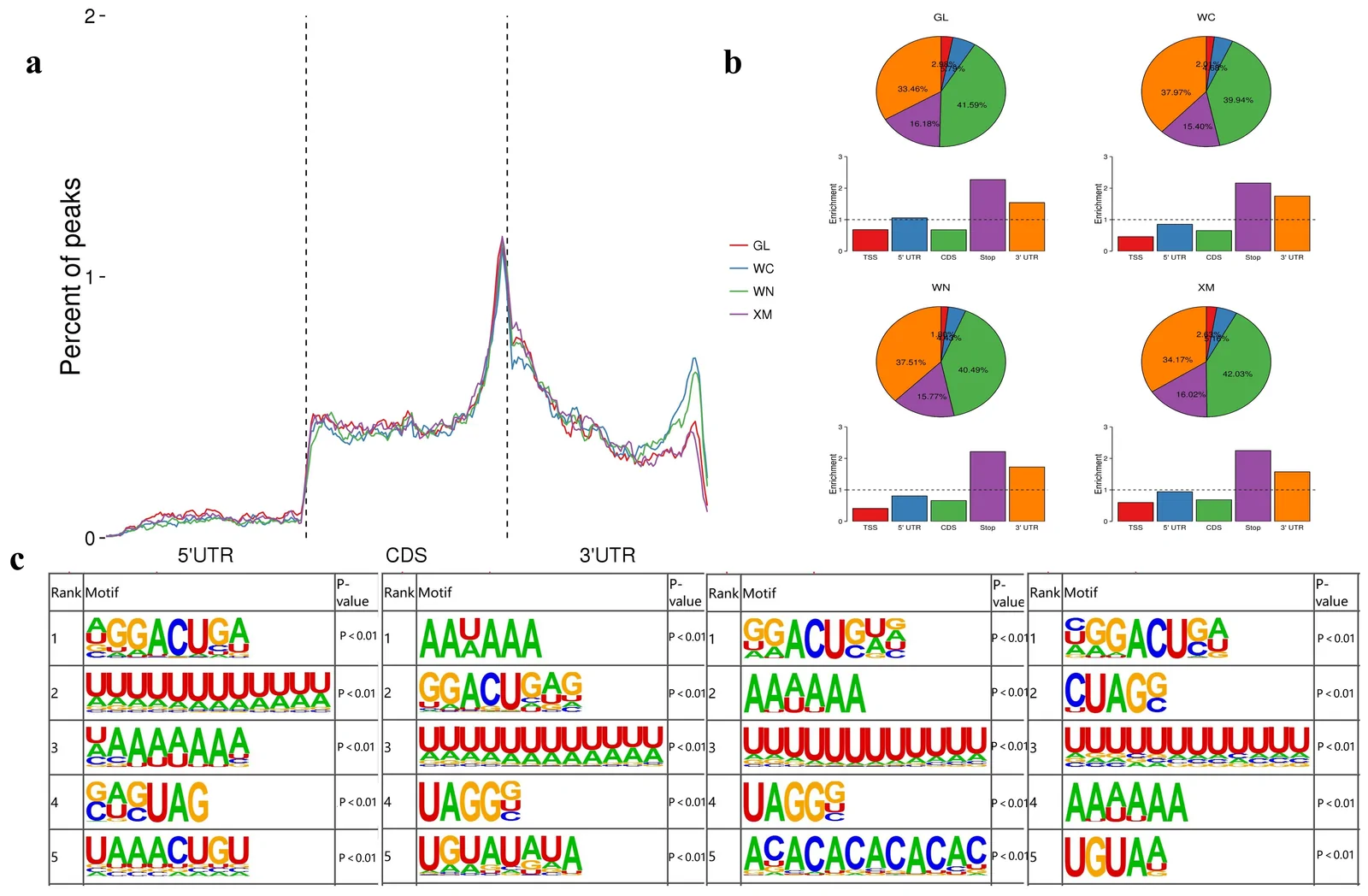
Overall characteristics of m6A enrichment in longissimus dorsi muscle. (**a**) Group-level aggregate metagene profiles of m6A peak density along transcripts; replicate-level confidence intervals were not generated. (**b**) Transcript-region distribution of m6A peaks among four cattle breeds. (**c**) enriched motifs compatible with the RRACH consensus.

To examine m6A enrichment relative to annotated transcript features, metagene profiles were compared among the four cattle groups. These profiles represent normalized positions within transcript models rather than chromosomal genomic coordinates. As shown in Figure 1a, the four groups displayed similar distribution patterns. m6A enrichment showed two main maxima: one near the boundary between the 5′ untranslated region (5′UTR) and the coding sequence (CDS), and the other near the stop codon and 3′UTR.

For regional annotation, each m6A-enriched region was assigned to one of five transcript-associated categories: the transcription start region, 5′UTR, CDS, stop-codon region, or 3′UTR. More than 40% of the m6A-enriched regions were assigned to the CDS, and more than 33% were assigned to the 3′UTR. Less than 3% were assigned to the transcription start region (Figure 1b). Motif analysis of the identified peak regions was performed using HOMER. The top five enriched m6A motifs in each group are shown in Figure 1c. Several enriched motifs were compatible with the canonical RRACH consensus sequence (R = A/G, H = A/C/U). Among the reported motifs, GGACU and AGACA matched this consensus. Their enrichment supports the expected sequence context of m6A-enriched regions but does not independently confirm m6A modification identity.

### 3.2. Differential m6A Modification Between GL and XM Cattle

**Figure 2:**
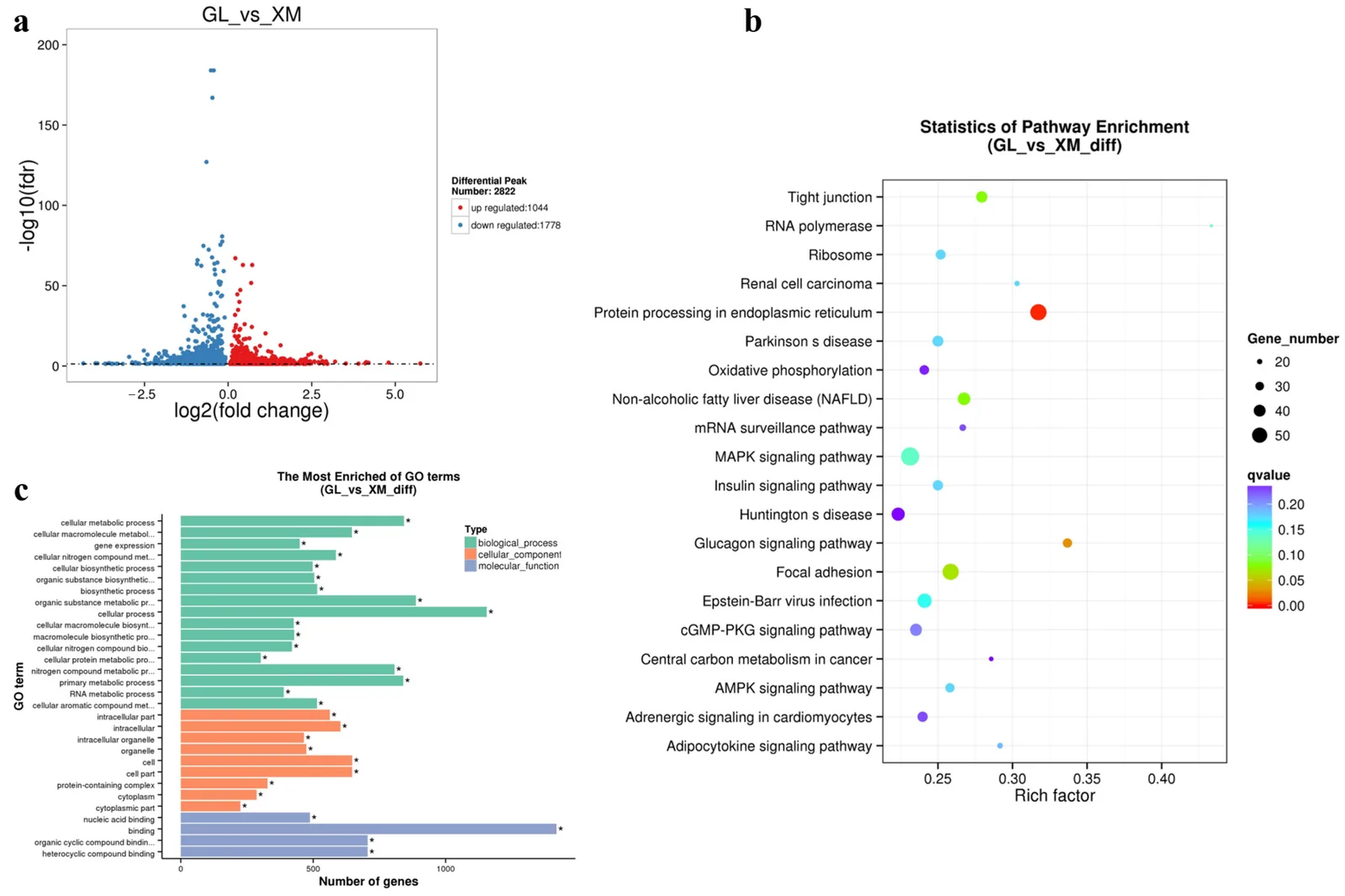
Differential m6A methylation modification between the GL and XM groups. (**a**) Hypermethylated and hypomethylated differentially enriched m6A peaks between the GL and XM groups identified using FDR < 0.05 and FC = 1. A total of 2822 differentially enriched m6A peaks were identified, including 1044 hypermethylated and 1778 hypomethylated peaks in GL relative to XM. (**b**) Top-ranked KEGG pathways for genes associated with regions showing differential m6A enrichment. Only pathways with corrected *p* < 0.05 were considered statistically significant. (**c**) Top 30 significantly enriched GO terms; the *x*-axis represents the number of genes annotated to each GO term.

To compare m6A enrichment between Guizhou cattle and XM cattle, GL cattle were first compared with XM cattle. A total of 2822 differentially enriched m6A peaks were identified in GL cattle relative to XM cattle, including 1044 hypermethylated and 1778 hypomethylated peaks (FDR < 0.05, FC = 1; Figure 2a). Functional annotation was then performed for genes associated with differentially enriched m6A peaks. Gene Ontology (GO) and Kyoto Encyclopedia of Genes and Genomes (KEGG) pathway analyses were conducted for these genes.

KEGG analysis of genes associated with regions showing differential m6A enrichment between GL and XM identified two pathways that remained significant after multiple-testing correction: protein processing in the endoplasmic reticulum (corrected *p* = 0.0026) and the glucagon signaling pathway (corrected *p* = 0.0296) (Figure 2b). Representative genes contributing to these enrichment results included *HSPA5* for protein processing in the endoplasmic reticulum and *AKT2* and *PPARGC1A* for glucagon signaling. The top 30 enriched GO terms ranked by corrected *p*-value are shown in Figure 2c. In the biological process category, these genes were mainly associated with organic substance metabolism, primary metabolism, nitrogen compound metabolism, and cellular metabolism.

### 3.3. The m6A Modification Differences Between WC and XM

**Figure 3:**
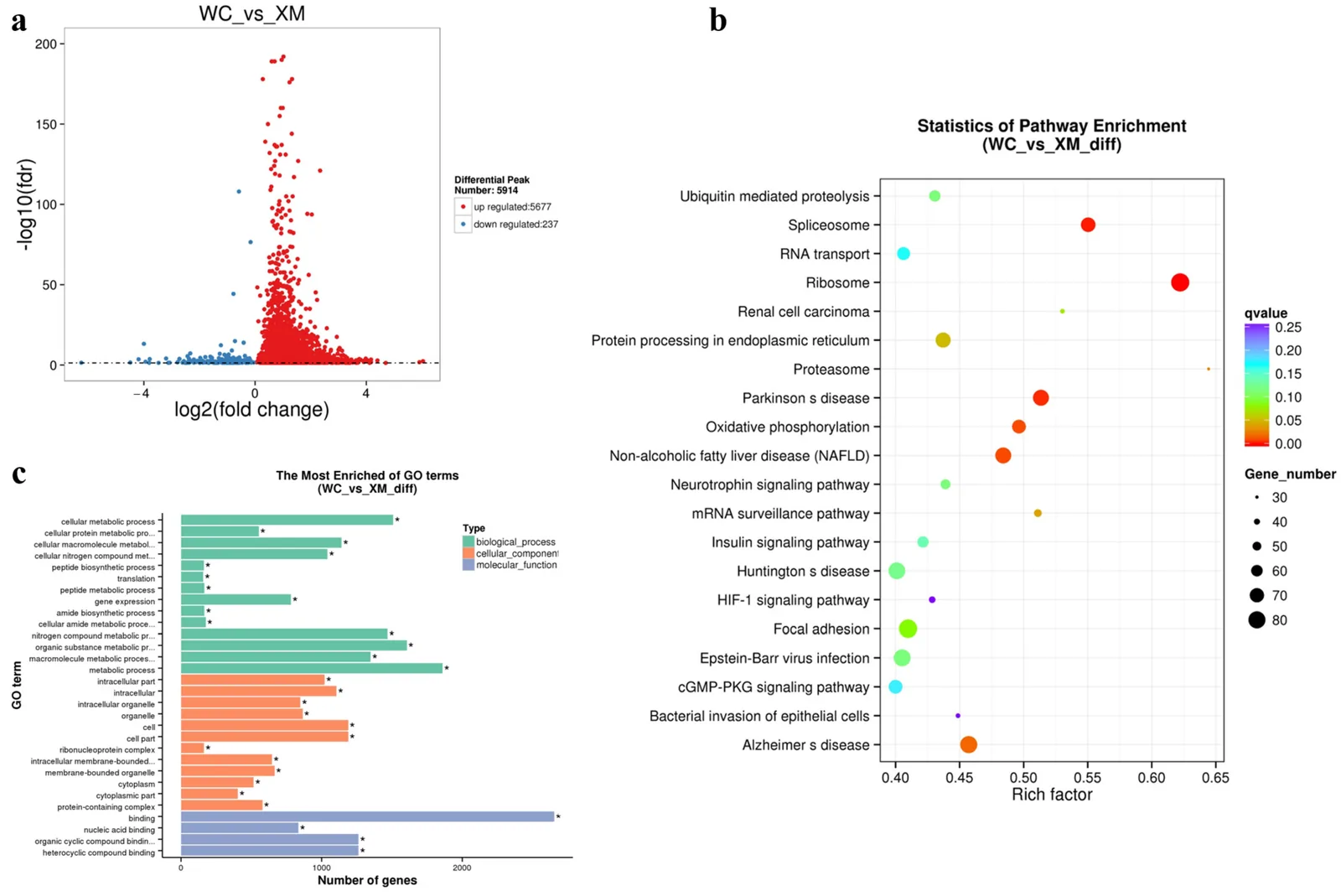
Differential m6A methylation modification between the WC and XM groups. (**a**) Hypermethylated and hypomethylated differentially enriched m6A peaks between the WC and XM groups identified using FDR < 0.05 and FC = 1. A total of 5914 differentially enriched m6A peaks were identified, including 5677 hypermethylated and 237 hypomethylated peaks in WC relative to XM. (**b**) KEGG pathway enrichment analysis of genes associated with differentially enriched m6A peaks; the *x*-axis represents the rich factor, calculated as the ratio of differentially enriched m6A peak-associated genes to all genes annotated in the corresponding pathway. (**c**) Top 30 significantly enriched GO terms; the *x*-axis represents the number of genes annotated to each GO term.

WC cattle were compared with XM cattle to characterize differential m6A enrichment. A total of 5914 regions showing differential m6A enrichment were identified in WC relative to XM, including 5677 regions with increased enrichment and 237 regions with decreased enrichment (FDR < 0.05, FC = 1; Figure 3a). KEGG analysis identified significant enrichment in the ribosome, spliceosome, oxidative phosphorylation, and the KEGG-labeled non-alcoholic fatty liver disease (NAFLD) pathways (corrected *p* = 9.96 × 10^−6^, 0.0014, 0.0094, and 0.0093, respectively; Figure 3b). The NAFLD annotation showed substantial overlap with mitochondrial energy metabolism and metabolic signaling genes. Forty annotated genes in this category were also assigned to oxidative phosphorylation. Representative genes included *NDUFS1*, *UQCRC2*, and *AKT2*. In skeletal muscle, this enrichment was interpreted as reflecting mitochondrial energy metabolism and metabolic signaling rather than liver-disease relevance. The top 30 enriched GO terms ranked by corrected *p*-value are shown in Figure 3c. In the biological process category, these genes were mainly associated with organic substance metabolism, nitrogen compound metabolism, cellular nitrogen compound metabolism, and cellular metabolism.

### 3.4. Differential m6A Modification Between WN and XM Cattle

**Figure 4:**
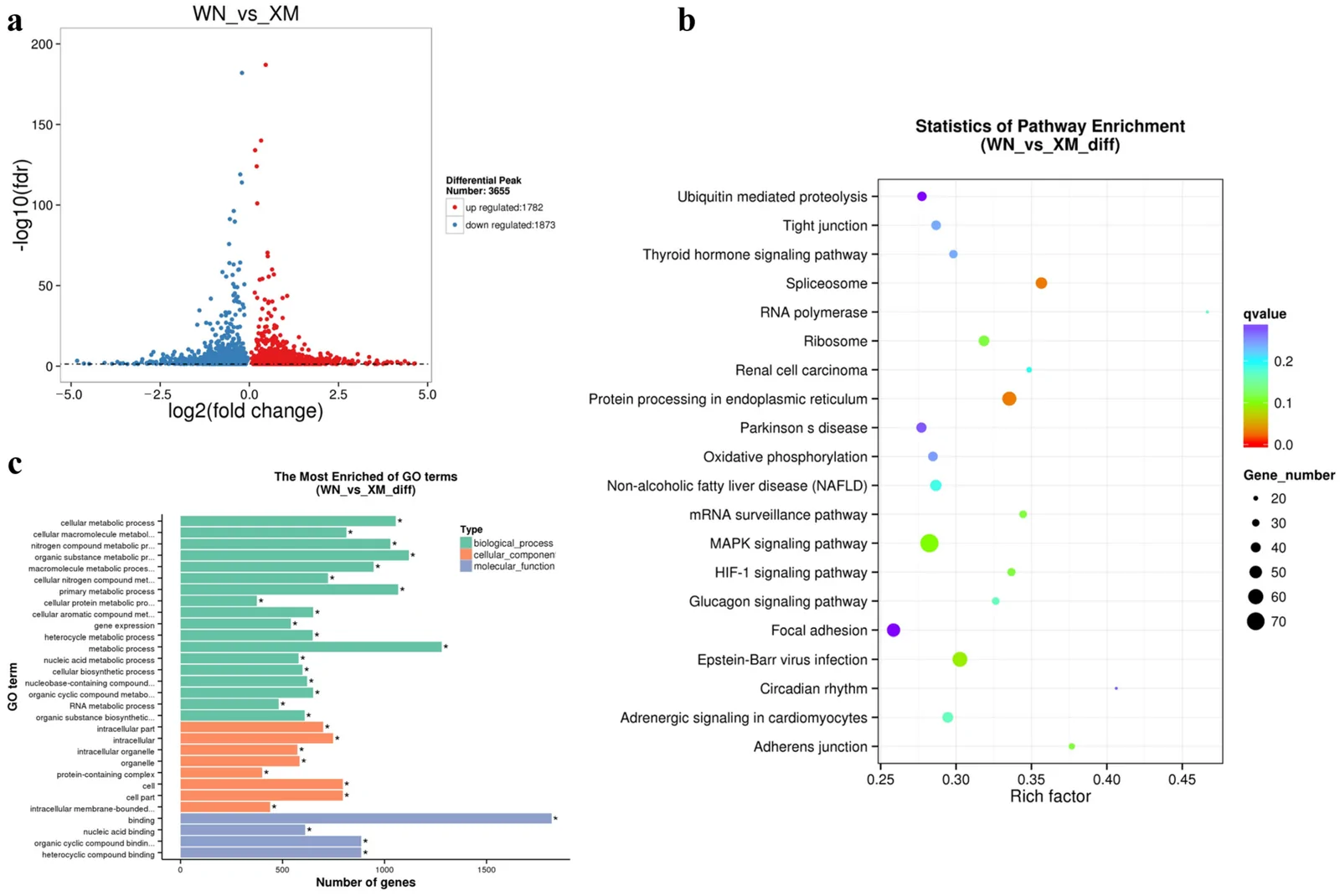
Differential m6A methylation modification between the WN and XM groups. (**a**) Hypermethylated and hypomethylated differentially enriched m6A peaks between the WN and XM groups identified using FDR < 0.05 and FC = 1. A total of 3655 differentially enriched m6A peaks were identified, including 1782 hypermethylated and 1873 hypomethylated peaks in WN relative to XM. (**b**) KEGG pathway enrichment analysis of genes associated with differentially enriched m6A peaks; the *x*-axis represents the rich factor. (**c**) Top 30 significantly enriched GO terms; the *x*-axis represents the number of genes annotated to each GO term.

In WN cattle, 3655 differentially enriched m6A peaks were identified relative to XM cattle, including 1782 hypermethylated and 1873 hypomethylated peaks (FDR < 0.05, FC = 1; Figure 4a). KEGG analysis identified significant enrichment of genes associated with regions showing differential m6A enrichment in protein processing in the endoplasmic reticulum and the spliceosome (corrected *p* = 0.0282 for both pathways; Figure 4b). Representative genes contributing to these enrichment results included *HSPA5* and *HSPA8* for protein processing in the endoplasmic reticulum, and *HSPA8* and *PRPF8* for the spliceosome. (Figure 4b). The top 30 enriched GO terms ranked by corrected *p*-value are shown in Figure 4c. In the biological process category, these genes were mainly involved in organic substance metabolism, primary metabolism, cellular metabolism, and nitrogen compound metabolism.

### 3.5. Comparative Analysis of Differential m6A Peak Signals Among Guizhou Cattle Breeds

To further compare m6A methylation profiles among the three indigenous Guizhou cattle breeds, pairwise comparisons were performed among WN, WC, and GL cattle. Heatmaps of normalized m6A enrichment signals showed distinct differential peak patterns in the WN vs. WC, WC vs. GL, and WN vs. GL comparisons (Supplementary Figure S1). In each comparison, subsets of differentially enriched m6A peaks showed higher signals in one breed than in the other, indicating breed-associated variation in m6A enrichment among Guizhou cattle breeds.

Motif enrichment analysis was also performed for differential m6A peak regions identified in the three intra-Guizhou comparisons. The enriched motifs in these differential peak regions showed sequence features compatible with m6A-associated motifs. This result supports the presence of breed-associated differential peaks in sequence contexts relevant to m6A modification. Together with the global m6A distribution shown in Figure 1, these results suggest that GL, WC, and WN cattle share broadly conserved m6A methylation patterns while retaining breed-specific m6A enrichment signatures. However, these intra-Guizhou differences should be interpreted as evidence of epitranscriptomic diversity rather than direct evidence of functional divergence, because further expression and functional validation are required.

### 3.6. Protein–Protein Interaction Network Analysis of Genes Associated with Differentially Enriched m6A Peaks

**Figure 5:**
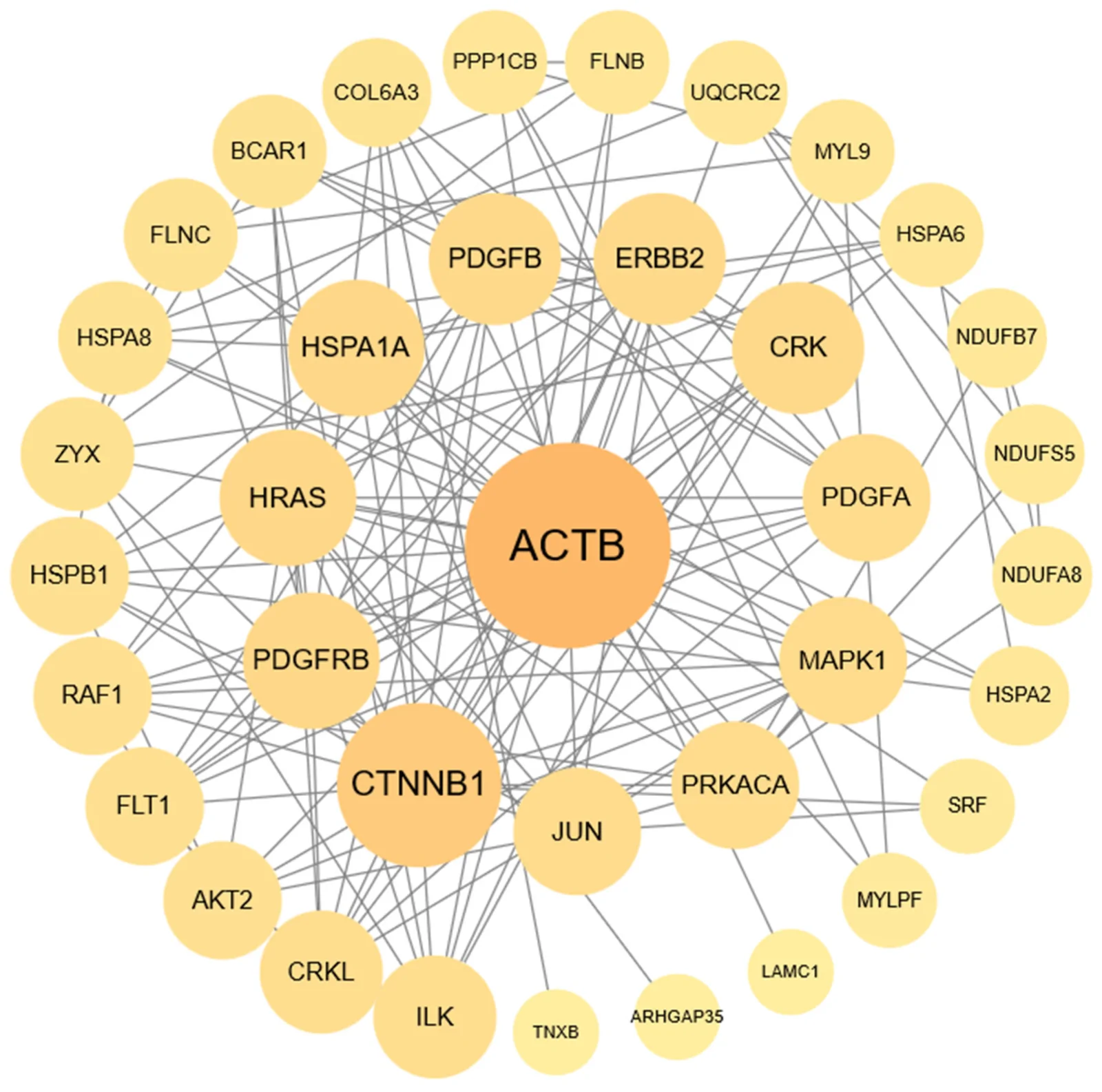
Database-inferred protein–protein interaction network of genes associated with differentially enriched m6A peaks. Interactions were obtained from STRING for Bos taurus and visualized in Cytoscape. Larger nodes indicate higher degree centrality. Labeled genes are high-connectivity candidate nodes for follow-up, not experimentally validated hubs.

To explore potential functional relationships among genes associated with differentially enriched m6A peaks, an inferred PPI network was constructed using the STRING database and visualized in Cytoscape. The input genes were protein-coding genes associated with differentially enriched m6A peaks identified in comparisons between Guizhou cattle and XM cattle (Figure 5). Because the interactions were obtained from public databases and were not experimentally measured in the longissimus dorsi muscle samples, this network should be interpreted as a hypothesis-generating framework rather than direct evidence of active protein interactions in bovine skeletal muscle.

Within the database-inferred network, ACTB, CTNNB1, JUN, MAPK1, and AKT2 showed high degree centrality and were prioritized as candidates for follow-up. Their ranking reflects network topology and existing STRING annotations rather than experimentally measured interactions. In particular, ACTB is a highly conserved, broadly connected cytoskeletal protein; its central position may be driven partly by extensive database annotation and should not be interpreted as evidence that ACTB is a causal regulator in these samples.

### 3.7. qRT-PCR Analysis of Candidate m6A-Associated Genes

**Figure 6:**
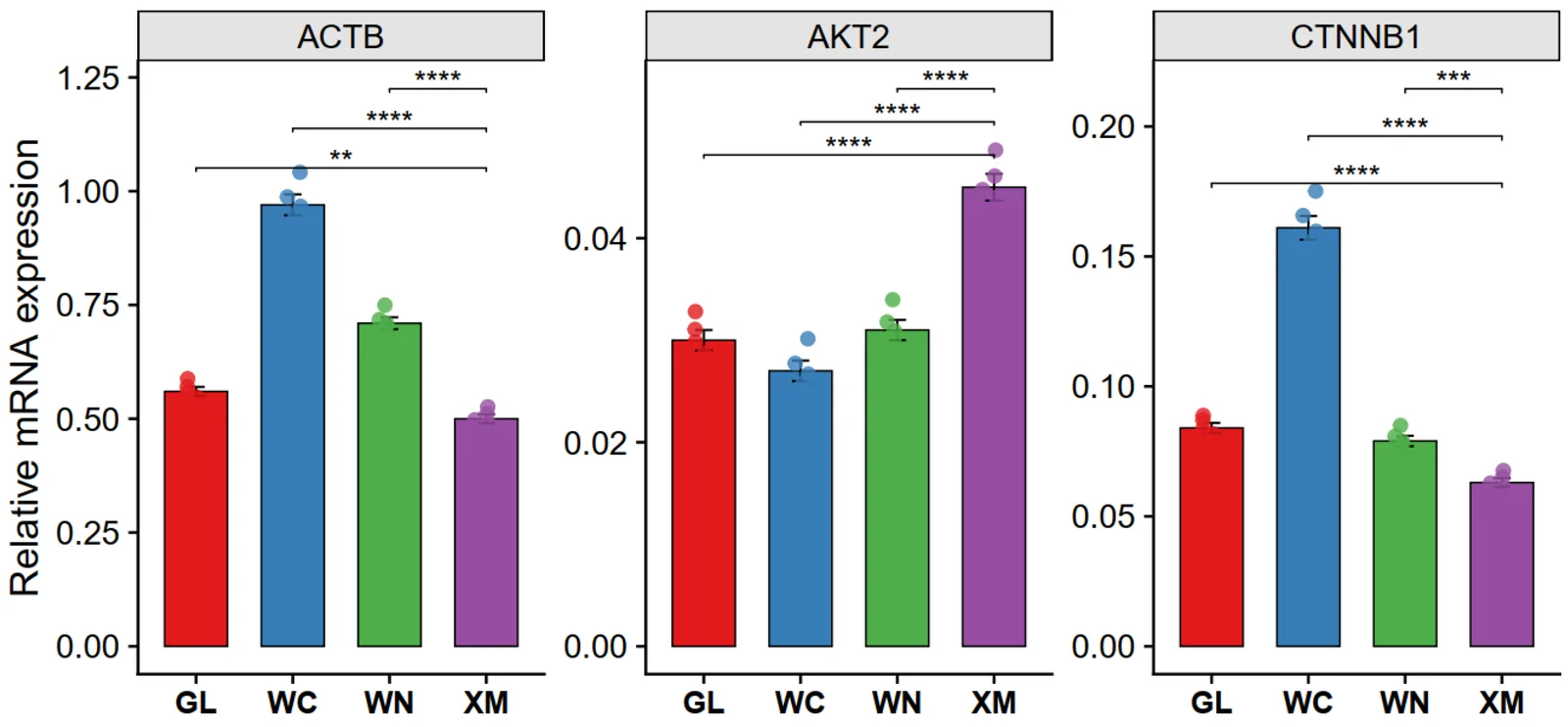
qRT-PCR analysis of candidate m6A-associated genes in longissimus dorsi muscle. Relative *ACTB*, *AKT2*, and *CTNNB1* transcript abundance was measured in GL, WC, WN, and XM cattle using *GAPDH* normalization. Individual data points for each group (n =5) are shown as dots overlaying the bars. Brackets and asterisks denote the displayed pairwise comparisons (** *p* < 0.01, *** *p* < 0.001, and **** *p* < 0.0001).

To determine whether selected genes associated with differentially enriched m6A peaks also showed breed-associated transcript differences, *ACTB*, *AKT2*, and *CTNNB1* mRNA abundance was measured by qRT-PCR (Figure 6). *ACTB* abundance was highest in WC cattle and lowest in XM cattle; GL and WN cattle also showed higher *ACTB* abundance than XM cattle. These measurements evaluate transcript abundance only and do not validate the corresponding m6A peaks.

*AKT2* showed the opposite expression pattern. *AKT2* transcript abundance was highest in XM cattle and was significantly lower in GL, WC, and WN cattle. Among the three indigenous breeds, WC cattle showed the lowest *AKT2* transcript abundance, whereas WN and GL cattle showed slightly higher levels that remained lower than those in XM cattle. *CTNNB1* transcript abundance also differed among breeds. WC cattle showed the highest *CTNNB1* transcript abundance, which was substantially higher than that in XM cattle. GL and WN cattle also showed higher *CTNNB1* transcript abundance than XM cattle, although the increase was smaller than that observed in WC cattle.

Overall, *ACTB* and *CTNNB1* transcript abundance was higher, whereas *AKT2* transcript abundance was lower, in the indigenous cattle groups than in XM cattle. These results demonstrate group-associated differences in steady-state transcript abundance under the sampled conditions. No conclusions were drawn regarding protein abundance, protein activity, pathway activity, or the relationship between transcript abundance and m6A enrichment.

## 4. Discussion

In this study, we characterized the transcriptome-wide m6A methylation landscape in longissimus dorsi muscle from three indigenous Guizhou cattle breeds, namely *Guanling*, *Wuchuan*, and *Weining* cattle, and compared these profiles with those of *Simmental* cattle. The results showed that m6A peak distribution was broadly conserved across groups, with predominant enrichment in the coding sequence (CDS) and 3′ untranslated region (3′UTR), particularly around the stop codon. This distribution pattern is consistent with previous mammalian studies showing that m6A peaks are commonly enriched near stop codons and within 3′UTRs [13,14]. Therefore, breed-associated differences were more likely reflected by variation in methylation intensity and gene-specific enrichment than by major changes in global m6A distribution.

This study provides a transcriptome-wide map of m6A-enriched regions in Guizhou and Simmental cattle and identifies breed-associated differential peaks, enriched pathways, and network-prioritized candidate genes. The data demonstrate differences at the level of regional m6A enrichment and selected transcript abundance. They do not establish a causal sequence from m6A modification to mRNA expression, protein activity, skeletal muscle phenotype, or meat quality.

MeRIP-seq measures antibody-enriched RNA regions rather than site-specific modification stoichiometry. Moreover, m6A effects are reader- and context-dependent: *YTHDF2* can promote transcript decay through CCR4–NOT recruitment [15], whereas *METTL3*-dependent modification can enhance translation in other settings [16,17]. Thus, hyper- or hypoenrichment cannot be translated directly into increased or decreased gene expression, and the differential peaks reported here represent candidate post-transcriptional regulatory regions.

The four breeds showed a conserved global distribution of m6A enrichment, together with marked differences in peak number and specific enriched regions. Pairwise comparisons among GL, WC, and WN also identified breed-associated peak patterns containing sequence motifs compatible with m6A modification. These results establish epitranscriptomic diversity among the sampled groups, while the biological effects of individual peaks remain to be tested.

Functional enrichment placed differentially peak-associated genes mainly in metabolic [18,19,20], RNA-processing, translational [21], mitochondrial, and signal-transduction contexts [22,23]. In the GL versus XM comparison, genes associated with differential m6A enrichment were significantly enriched in protein processing in the endoplasmic reticulum and glucagon signaling after multiple-testing correction. In the WC versus XM comparison, genes associated with regions showing differential m6A enrichment were significantly enriched in ribosome, spliceosome, oxidative phosphorylation, and the KEGG-labeled NAFLD pathway. The NAFLD annotation showed substantial overlap with oxidative phosphorylation and was interpreted as reflecting shared mitochondrial and metabolic signaling processes rather than evidence of liver disease in skeletal muscle.

The STRING/Cytoscape network was used only to prioritize candidate genes for subsequent investigation. It does not demonstrate active or direct protein–protein interactions in the sampled longissimus dorsi tissue. ACTB, CTNNB1, JUN, MAPK1, and AKT2 ranked highly by degree centrality and were therefore considered network-prioritized candidates. As an illustrative database-level example, the Bos taurus STRING v12.0 database reported a combined confidence score of 0.988 for the AKT2–PIK3CA association. This score indicates strong support within the STRING database but may include orthology-transferred evidence and does not confirm direct physical binding, cattle-specific interaction, or biological activity in the sampled muscle tissue. ACTB requires particular caution because its extensive database connectivity and fundamental cytoskeletal role may increase its network degree independently of context-specific biological importance.

*ACTB* showed differential m6A enrichment and transcript abundance, but neither ACTB protein abundance nor activity was measured. ACTB expression can vary during myogenic differentiation [24,25,26]. As a cytoskeletal protein, β-actin contributes to cellular structure and force transmission. Previous genomic work described Weining cattle as adapted to cold, humid mountainous environments and noted good climbing ability [27]. Thus, *ACTB*-associated m6A enrichment may reflect post-transcriptional adjustment of cytoskeletal maintenance under local locomotor demands. This interpretation remains hypothetical because terrain exposure, locomotor activity, ACTB protein turnover, and muscle mechanical properties were not measured. The present data therefore support *ACTB* only as a network-prioritized, m6A-associated candidate. Cytoskeletal proteins have also been associated with post-mortem muscle biology and tenderness-related traits [28,29,30].

*CTNNB1* participates in Wnt/β-catenin signaling and has documented associations with myogenic differentiation and intramuscular adipose biology [31,32,33,34]. Its differential m6A enrichment and transcript abundance identify *CTNNB1* as a biologically plausible candidate for breed-associated muscle regulation. However, the direction and functional consequence of the m6A-associated difference require direct methylation-site, protein, and cellular validation.

*AKT2* is a component of *PI3K–AKT* signaling involved in muscle metabolism, growth, and adipogenic regulation [35]. Lower *AKT2* transcript abundance in the indigenous breeds, together with differential m6A enrichment, prioritizes this gene for mechanistic study. The present design cannot determine whether the enrichment difference affects *AKT2* stability, translation, or downstream signaling.

qRT-PCR showed breed-associated differences in *ACTB*, *CTNNB1*, and *AKT2* transcript abundance, but it did not validate m6A enrichment. No MeRIP-qPCR, site-specific m6A assay, protein quantification, or perturbation of m6A writers, erasers, or readers was performed. Likewise, intramuscular fat, muscle-fiber characteristics, tenderness, and Warner–Bratzler shear force were not measured. Accordingly, literature-based links to muscle and meat-quality biology are used to motivate hypotheses, not to claim phenotype–epigenotype causality. Future studies should integrate RNA-seq and MeRIP-seq data from the same biological samples to test whether m6A enrichment is associated with steady-state transcript abundance. Because RNA-seq does not measure RNA half-life, direct RNA decay assays are still required to determine whether hypomethylated transcripts have altered stability.

The main limitation is the small MeRIP-seq cohort (three independent animals per breed), which provides limited power to characterize within-breed genetic heterogeneity, subtle effects, or population structure. The results should therefore be treated as exploratory and hypothesis-generating. All cattle were managed under grazing-based production conditions and fed conventional diets, which reduced major husbandry differences among groups. However, detailed individual feed composition and intake were unavailable. Dietary effects on m6A regulation have been reported in ruminants; rumen-protected methionine and lysine altered m6A levels and the expression of related enzymes in lamb liver and muscle [36]. Comparable evidence for conventional forage effects on m6A regulation in bovine skeletal muscle remains limited. Thus, the observed patterns are interpreted as breed-associated under comparable management, while minor environmental effects cannot be fully excluded. Larger, balanced cohorts sampled under matched management are required for confirmation.

Additional technical limitations should also be considered. MeRIP-seq detects regional m6A enrichment rather than precise modification sites or methylation stoichiometry. Although BWA-MEM has been used in published MeRIP-seq workflows, it is not a dedicated splice-aware aligner. Therefore, some exon–exon junction-spanning reads may have been underrepresented, and the present analysis should be interpreted as characterizing regional m6A enrichment rather than splice-junction- or isoform-specific modification patterns. Other limitations include the absence of numerical RIN values in the archived QC report; use of a single qRT-PCR reference gene without formal stability testing; lack of expression profiling for m6A writers (METTL3, METTL14, WTAP), erasers (FTO, ALKBH5), and readers (YTHDF1/2/3, YTHDC1/2); and absence of MeRIP-qPCR, site-specific methylation, protein-level, and functional validation. Future studies should integrate validated multi-gene qRT-PCR normalization, regulator-expression analysis, MeRIP-qPCR or site-resolved assays, proteomics, functional perturbation, and measured meat-quality traits. Because m6A readers shape the fate of methylated transcripts, breed-associated differences in YTHDF1, YTHDF2, or YTHDF3 could alter the functional consequences of similar m6A enrichment patterns [17]. Future studies should quantify these readers in the same muscle samples and integrate their expression with MeRIP-seq, RNA-seq, and protein-level data.

## 5. Conclusions

In summary, this study maps group-level m6A enrichment in longissimus dorsi muscle from three indigenous Guizhou cattle breeds and Simmental cattle. The four groups shared a broadly conserved transcript distribution, while differing in peak number, differential enrichment, associated pathways, and database-inferred network topology. *ACTB*, *CTNNB1*, and *AKT2* also showed breed-associated transcript-abundance differences and are prioritized for targeted follow-up.

These results constitute an exploratory epitranscriptomic resource rather than evidence that m6A differences cause gene-expression changes or meat-quality phenotypes. Confirmation will require larger cohorts, replicate-resolved analyses, measured phenotypes, validated reference-gene normalization, profiling of m6A regulatory machinery, MeRIP-qPCR or site-specific assays, protein measurements, and functional experiments.

## Figures and Tables

**Table 1:** Statistics and quality control of data generated by MeRIP sequencing.

Sample	Raw_Reads	Clean_Reads	Clean_Ratio	GC_Content	Q20_Clean	Q30_Clean
GL_input	22,908,563	22,704,625	89.23%	52.26%	97.63%	93.37%
GL_ip	20,897,897	20,688,672	90.75%	52.55%	97.41%	92.88%
WC_input	24,370,553	24,180,786	87.96%	45.19%	97.50%	92.86%
WC_ip	21,315,435	21,164,130	90.61%	45.89%	97.90%	93.74%
WN_input	22,302,860	22,079,897	88.34%	51.23%	97.74%	93.64%
WN_ip	25,271,768	24,996,830	90.50%	50.87%	97.67%	93.49%
XM_input	22,969,853	22,776,447	88.82%	52.65%	97.61%	93.24%
XM_ip	22,574,540	22,346,298	91.14%	53.02%	97.49%	93.08%

**Table 2:** Statistics of mapping data.

Sample	Clean_Reads	rRNA	non_rRNA	Mapped	Unique_Mapped
GL_input	22,704,625	213,935 (0.94%)	22,490,690	21,180,444 (94.17%)	20,572,779 (97.13%)
GL_ip	20,688,672	174,587 (0.84%)	20,514,085	19,224,581 (93.71%)	18,689,580 (97.22%)
WC_input	24,180,786	1,437,641 (5.95%)	22,743,145	21,668,960 (95.28%)	21,304,547 (98.32%)
WC_ip	21,164,130	996,621 (4.71%)	20,167,509	19,251,315 (95.46%)	18,783,771 (97.57%)
WN_input	22,079,897	411,928 (1.87%)	21,667,969	20,603,871 (95.09%)	19,999,662 (97.07%)
WN_ip	24,996,830	368,946 (1.48%)	24,627,884	23,336,185 (94.76%)	22,563,812 (96.69%)
XM_input	22,776,447	223,710 (0.98%)	22,552,737	21,140,802 (93.74%)	20,530,182 (97.11%)
XM_ip	22,346,298	169,835 (0.76%)	22,176,463	20,527,779 (92.57%)	19,985,224 (97.36%)

**Table 3:** Summary of group-level m6A peaks identified in each cattle breed.

Group	Fragment Length	Count of Peak	FRIP
GL	156	17,659	53.8100%
WC	151	19,530	57.4155%
WN	154	17,501	53.0528%
XM	157	16,271	55.1337%

Note: FRIP (fraction of reads in peaks) is the percentage of uniquely mapped immunoprecipitation reads overlapping called m6A peaks. FRIP values ranged from 53.0528% to 57.4155% across breeds and were interpreted as within-study enrichment metrics.

## Data Availability

The data presented in this study are available from the corresponding author upon reasonable request.

## Supplementary Materials

The following supporting information can be downloaded at: https://www.mdpi.com/article/10.3390/life16081252/s1. Table S1: The primer sequences used for qPCR. Figure S1: Differential m6A peak-signal heatmaps and motif enrichment among WN, WC, and GL cattle. Heatmap rows represent differentially enriched peak regions and columns represent breed groups. Colors show row-scaled normalized enrichment signals, with red indicating relatively higher and blue indicating relatively lower signal; the scale does not represent absolute methylation percentage. Sequence logos show the top enriched motifs for each pairwise comparison.
