# Supplementary material for: Transcriptome-Wide m6A Methylation Landscape of Longissimus Dorsi Muscle in Indigenous Guizhou Cattle
Source: Life (Basel). 2026 Jul 29;16(8):1252. doi: 10.3390/life16081252 (PMC13514678; doi:10.3390/life16081252)
Supplement: Supplementary file 1 [file life-16-01252-s001.zip › life-4403694-supplementary.pdf]

Supplementary material:

Table S1： The primer sequences used for qPCR.

Gene	Primer sequences	Product size	Primer temperature
GAPDH	F: CACCCTCAAGATTGTCAGCAATGCC<br>R: ATGGCGTGGACAGTGGTCATAAGTC	117 bp	59.3/59.3 °C
ACTB	F: GGAAGTCCTTTGCCTTCCCAAAAGC<br>R: ACAAAAGCGATCACCTCCCCTGTG	97 bp	59.3/59.1 °C
CTNNB1	F: CCAATGGCTTGGAATGAGACTGCTG<br>R: TAGCTGGGATCATCCTGGCGATATC	89 bp	59.3/59.3 °C
AKT2	F: CTGTCGCAGAATGCCAGCTGATG<br>R: ATCCACTCTTCCCTCTCGTCTGG	133 bp	58.8/58.8 °C

Figure S1：

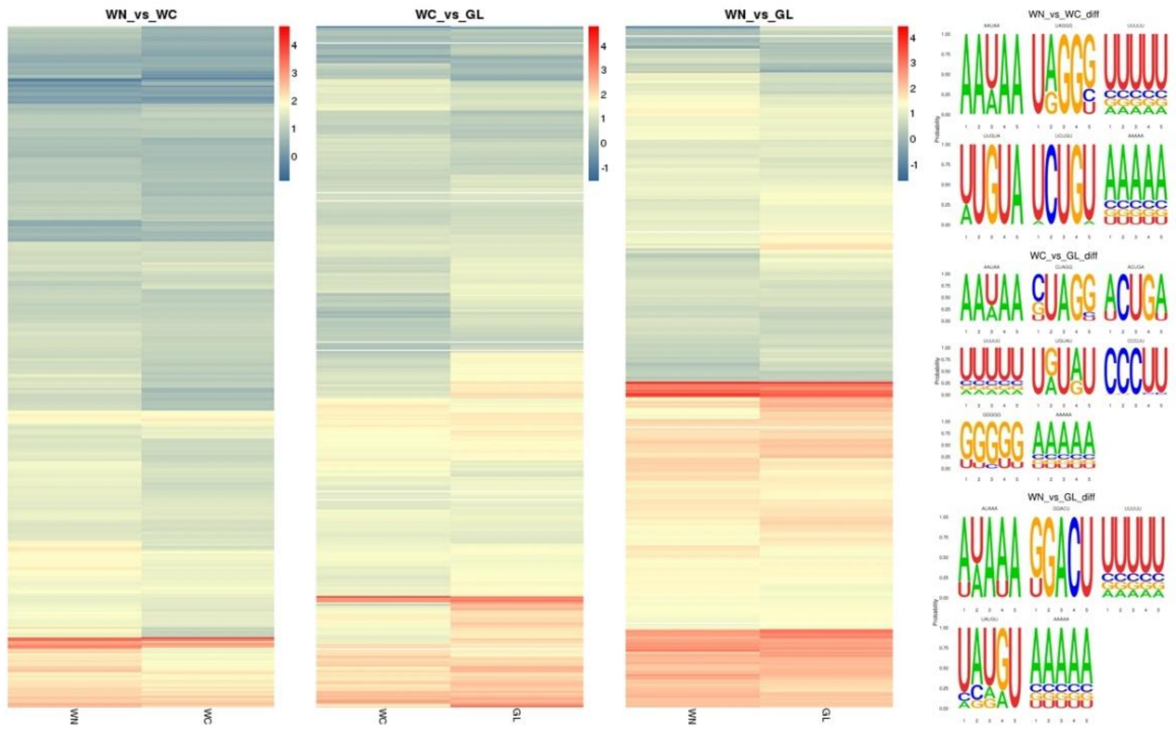

Supplementary Figure S1. Differential m6A peak-signal heatmaps and motif enrichment among WN, WC, and GL cattle. Heatmap rows represent differentially enriched peak regions and columns represent breed groups. Colors show row-scaled

normalized enrichment signals, with red indicating relatively higher and blue indicating relatively lower signal; the scale does not represent absolute methylation percentage. Sequence logos show the top enriched motifs for each pairwise comparison.
